# Identification of important outcomes for surgical and brace treatment of adolescent idiopathic scoliosis: qualitative analysis of the perspectives of young people, parents, and healthcare professionals

**DOI:** 10.1302/2633-1462.71.BJO-2025-0218.R1

**Published:** 2026-01-12

**Authors:** Lisa Graham-Wisener, Samuel Sloan, Julie McMullan, Rebecca Waterworth, Ciara Close, Mike Clarke, Robin Gordon, Paul Toner

**Affiliations:** 1 School of Psychology, Queen’s University Belfast, Belfast, UK; 2 Musgrave Park Hospital, Belfast Health and Social Care Trust, Belfast, UK; 3 School of Nursing & Midwifery, Queen’s University Belfast, Belfast, UK; 4 Centre for Public Health, Queen’s University Belfast, Block A, Royal Victoria Hospital, Belfast, UK; 5 School of Health Sciences, University of Dundee, Dundee, UK

**Keywords:** Adolescent idiopathic scoliosis, AIS, Qualitative, Treatment outcome, Core outcome set, Perspective, Experience, clinical trials, healthcare professionals, clinical outcomes, brace treatment, clinicians, Scoliosis, Patient-reported outcome measures (PROMs), surgical complications, Cobb angle

## Abstract

**Aims:**

High-quality clinical trials in adolescent idiopathic scoliosis (AIS) are needed to guide decision-making but progress is hindered by suboptimal selection of outcome measures. Identifying meaningful outcomes for consistent measurement across clinical trials and routine practice is critical. However, there is currently no understanding of which treatment outcome domains are considered important by adolescents, their parents, and healthcare professionals (HCPs). This study is the first to address this gap internationally.

**Methods:**

This study represents the first stage of core outcome set (COS) development, following gold-standard guidance. A cross-sectional qualitative interview study with 40 participants (adolescents with AIS, their parents, and HCPs) was conducted. Semi-structured interviews were analyzed to identify and categorize important AIS treatment outcomes. Analytical rigour was ensured through coder agreement and stakeholder consultation.

**Results:**

A total of 91 important outcome domains were identified; 53 outcome domains applying to both bracing and surgery, with 15 additional outcome domains for bracing only, and 23 additional outcome domains for surgery only. Of the 91 outcome domains, more than three-quarters (71/91, 78%) related to life impact, with smaller proportions relating to physiological/clinical outcomes (13/91, 14%), resource use (4/91, 4%), and adverse events (1/91, 1%).

**Conclusion:**

The current study highlights treatment outcomes considered important by adolescents with AIS, their parents, and HCPs. These findings will inform outcome selection in clinical trials and routine practice, as well as facilitating an ongoing programme of research to develop a COS for evaluating treatment of AIS.

Cite this article: *Bone Jt Open* 2026;7(1):54–65.

## Introduction

Adolescent idiopathic scoliosis (AIS) is a progressive curvature of the spine with unknown pathology, affecting 1% to 3% of adolescents worldwide.^1^ Management of AIS varies internationally, with uncertainties around optimal bracing concepts, surgical approaches, and treatment indications.^2-5^ High-quality clinical trials are needed to guide decision-making,^6^ but progress is hindered by the suboptimal selection of outcome measures; applicability and inconsistency of outcomes measured,^7^ and the quality of measurement tools.^8^

Research is dominated by a focus on curve progression and radiological outcomes.^9^ Patient-reported outcome measures (PROMs), which capture patient perspectives, are essential for balanced clinical decision-making but are underused or narrowly focused on domains like pain and function.^10^ Ensuring the most important outcome domains are measured for the target population is a priority for clinicians, young people with AIS, and their families.^4^

A core outcome set (COS) for evaluating treatment of AIS, for which surgery and bracing are the main treatment types internationally, would help resolve the measurement problem. COSs are an agreed standard set of outcomes that should be measured and reported in all trials or in routine practice in specific areas of healthcare internationally.^11^ While additional outcomes may still be studied, a COS ensures consistent focus on priority outcomes, enhancing the cumulative value of research for evidence-based care. A COS for AIS would address international concerns by ensuring the most important outcomes are measured consistently.^7,12^ It would also promote the use of psychometrically robust measurement tools and encourage the development of new measures where needed.

Initial efforts to develop an AIS COS, such as a systematic review and a consensus process with Nordic spinal surgeons,^13^ lacked input from patients and the multidisciplinary team. Problems can also arise if a COS is developed through reliance solely on existing research,^13^ because the outcome domains would reflect earlier researchers' priorities and, potentially, fail to match the priorities of those involved in decision-making outside research.^14^ Advances in COS methodology now emphasize including diverse perspectives, particularly those with lived experience of a condition;^15,16^ however, currently, there is insufficient understanding of which treatment outcomes are important to adolescents with AIS, their parents, and healthcare professionals (HCPs). This study is a critical step towards aligning AIS treatment evaluation with the priorities of these groups, addressing barriers to global health policy and practice.

This study is part of the SPINE-COS-AYA research programme,^17^ which has the overall goal of developing an internationally applicable, person-centred COS for evaluating AIS treatment (bracing and surgery) in research and clinical practice. Minimum outcomes will be agreed upon for both treatment types, with additional outcomes specific to one of these interventions only. This first phase of research involves engaging adolescents with AIS, their parents, and HCPs, with the aim of understanding which treatment outcomes are important from their perspective. This will be used to generate a longlist of candidate outcome domains, which will then inform a subsequent international consensus process to agree a COS.

## Methods

### Study design

This is a cross-sectional qualitative interview study. Qualitative research helps uncover which outcomes are important and how stakeholders' perceptions of successful outcomes may differ.^14,18^

The research adhered to internationally recognized gold-standard guidelines for COS development outlined in the COMET Handbook and COS-STAD,^15,16^ with reporting in accordance with the COS-STAR statement.^19^ The study was pre-registered with COMET and the Open Science Framework.

Patient and public involvement was embedded throughout the study via the formation of a Young Person Advisory Panel (YPAG), ensuring that the perspectives of young people informed each stage.^18^

### Setting

Participants were recruited via the Northern Ireland Regional Scoliosis Service (UK).^20^

### Participants and recruitment

Three participant groups were included:

1. Adolescents and young adults (AYAs) diagnosed with AIS at aged ≥ ten years, not secondary to other conditions;
2. Parents or guardians of eligible AYAs; and
3. HCPs experienced in AIS care.

Maximum variation sampling ensured diverse perspectives, including AYAs at different treatment stages (zero to two and two to five years post-surgery, and conservative treatment-only), male/female participants, and a variety of ages (ten to 25 years). Parents of post-surgery and conservatively treated AYAs and HCPs from various disciplines were also included.

Recruitment used two approaches: 1) direct contact via outpatient clinics or letters following eligibility screening; and 2) study posters shared in clinics and on social media, whereby the AYA/parent contacted the researcher who then confirmed eligibility. Parents of AYAs aged ≥ 16 years were approached with the AYA’s consent. HCP participants were identified and approached directly.

### Data collection

Interviews were conducted via Teams (Microsoft, USA)/telephone or face-to-face. AYAs aged ≤ 18 years could opt for a parent to be present.^21^ Participants completed sociodemographic forms, with additional medical data extracted from records. Semi-structured interview guides for each participant were developed (see Supplementary Material for AYA example), informed by the AIS/COS research literature, and through consultation with the YPAG.

Interviews were undertaken by two postdoctoral researchers (JM, CC), experienced with qualitative methods and with no prior relationship to participants. Interviews lasted a mean 28 minutes (SD 9.5), were audio-recorded, and transcribed verbatim.

### Analysis

Data analysis involved qualitative inductive content analysis,^22^ in line with other COS development studies,^23,24^ using semantic and latent coding to identify explicit and inferred outcomes. Codes were generated directly from participant data rather than applying pre-defined categories. In line with COMET guidelines,^16^ the wording was extracted verbatim. Outcomes were defined as perceived consequences or impacts (positive/negative) of AIS treatment. Coding was performed independently by three researchers (LGW, PT, JMcM) with varied expertise. Separate analyses were conducted for each participant group, with similar codes merged to produce three tables of candidate outcome domains and verbatim quotes. Outcome domains were segregated into three groups: 1) outcome domains recorded across treatment types; 2) outcome domains recorded for bracing only; and 3) outcome domains recorded for surgery only.

The three tables of important outcome domains were scrutinized by the research team. We aimed for a high level of granularity throughout the analysis to avoid missing domains.^25^ The YPAG validated the interpretations and descriptors. Finally, the three tables of important outcome domains were consolidated in one long list and aligned to a trial outcome taxonomy.^26^

### Ethical approval

UK Health Research Authority Research Ethics Committee approval was obtained (REC Reference 20/WA/0088) and informed consent (with additional assent where applicable) obtained from all participants.

## Results

In total, 40 interviews were undertaken between June 2021 and August 2022: 18 with AYAs; nine with parents; and 13 with HCPs. The AYAs interviewed were largely female, but diverse in relation to other important characteristics such as age, time since diagnosis, and treatment received (Table I).

**Table I.** Adolescents and young adult participant sociodemographic and medical characteristics (n = 18).

Variable	Data
Mean age, yrs (SD; range)	16.8 (2.84; 12 to 22)
**Sex, n (%)**	
Female	15 (83.3)
Male	3 (16.7)
**Ethnicity, n (%)**	
White	18 (100)
**Main activity, n (%)**	
In school	13 (72.2)
At university	2 (11.1)
Employed	1 (5.6)
Not in education, training, or employment	2 (11.1)
**Comorbidities, n (%)**	
None	17 (94.4)
Two	1 (5.6)
**Treatment type, n (%)**	
Bracing	6 (33.3)
Surgery	12 (66.6)
**Surgery only participants (n = 12)**	**Data**
**Surgery type, n (%)**	
Posterior spinal fusion with instrumentation	12 (100)
**Time since surgery, n (%)**	
0 to 2 years	5 (41.7)
2 to 5 years	7 (58.3)
**Mean days in hospital, days (SD; range)**	7.70 (4.19, 5 to 19)
**Complications, n (%)**	
None	12 (100)
**Repeat surgery, n (%)**	
None	11 (91.7)
Awaiting costoplasty	1 (8.3)
**Previous bracing, n (%)**	
No	8 (66.6)
Yes	4 (33.3)
**Bracing only participants (n = 6)**	**Data**
**Brace treatment, n (%)**	
Current bracing	2 (33.3)
Previous bracing	4 (66.6)
**Brace type, n (%)**	
Full time rigid bracing	4 (66.6)
Not recorded	2 (33.3)
**Complications, n (%)**	
None	6 (100)
**Physiotherapy involvement, n (%)**	
No	4 (66.6)
Yes	2 (33.3)

The parents interviewed were largely mothers of AYAs, with only one father (Table II). The HCPs were experienced clinicians representing the main disciplines on the AIS multidisciplinary team (Table III).

**Table II.** Parent participant sociodemographic characteristics (n = 9).

Variable	N (%)
**Age, yrs**	
35 to 44	1 (11.1)
45 to 54	8 (88.9)
**Sex**	
Female	8 (88.9)
Male	1 (11.1)
**Ethnicity**	
White	9 (100)
**Highest education**	
NVQ	1 (11.1)
GSCEs	1 (11.1)
Vocational qualification	1 (11.1)
Diploma level 3	1 (11.1)
Undergraduate degree	2 (22.2)
Postgraduate degree	3 (33.3)
**Employment**	
Employed	7 (77.8)
Student/in training	1 (11.1)
Retired	1 (11.1)
**Marital status**	
Married	8 (88.9)
Unknown	1 (11.1)
**Relationship to young person**	
Mother	7 (77.8)
Stepmother	1 (11.1)
Father	1 (11.1)
**Living arrangement**	
With young person	9 (100)

GCSE, general certificate of secondary education; NVQ, national vocational qualification.

**Table III.** Healthcare professional participant sociodemographic characteristics (n = 13).

Variable	N (%)
**Age, yrs**	
25 to 34	2 (15.4)
35 to 44	4 (30.8)
45 to 54	7 (53.8)
**Sex**	
Female	10 (76.9)
Male	3 (23.1)
**Ethnicity**	
White	12 (92.3)
Other	1 (7.7)
**Highest education**	
Vocational qualification	2 (15.4)
Degree	6 (46.2)
Postgraduate degree	5 (38.5)
**Professional background**	
Medical professional (i.e. spinal surgeon, anaesthetist)	5 (38.5)
Registered nurse (i.e. staff nurse, specialist nurse)	4 (30.8)
Occupational therapist	1 (7.7)
Physiotherapist	3 (23.1)
**Years in role**	
Less than 2	1 (7.7)
3 to 5	1 (7.7)
6 to 9	3 (23.1)
10 +	8 (61.5)
**Experience with AIS, yrs**	
Less than 2	3 (23.1)
3 to 5	3 (23.1)
6 to 9	2 (15.4)
10 +	5 (38.5)

AIS, adolescent idiopathic scoliosis.

The interviews generated a total of 91 important outcome domains: 53 outcome domains applying to both treatment types, with 15 additional outcome domains for bracing only, and 23 additional outcome domains for surgery only (Table IV). The outcome domains identified ranged in level of specificity from single (e.g. curve progression) to multifaceted constructs (e.g. health-related quality of life). Most of the outcome domains (n = 54, 59%) were identified by at least two participant groups, suggesting a good level of agreement between AYAs, parents, and HCPs in relation to which outcomes are important to measure. A smaller number of additional outcome domains were identified only by healthcare professionals (n = 17, 19%), e.g. cardiac function, by parents (n = 10, 11%), e.g. repeat surgery, or by AYAs (n = 10, 11%), e.g. ability to maintain a safe pregnancy in future. Overall, HCPs identified the largest number of important outcome domains (n = 68, 75% of 91), followed by AYAs (n = 57, 63%), and then parents (n = 53, 58%).

**Table IV.** Combined important outcome domain longlist from qualitative interviews (n = 40).

Dodd’s et al^26^ taxonomy core area	Dodd’s et al core outcome domain	AIS outcome domain	AYA	HCP	Parents	Longlist of outcome domains in existing COS and related systematic review^21^*
**Outcomes relating to both bracing and surgery**						
**Physiological/clinical**	Musculoskeletal and connective tissue outcomes	Curve progression	**✓**	**✓**	**✓**	Curve progression
	General disorder	Pain	**✓**	**✓**	**✓**	Pain intensity/comfort
		Impact of pain	**✓**	**✓**	**✓**	-
		Fatigue	-	**✓**	**✓**	-
	Respiratory, thoracic, and mediastinal outcomes	Pulmonary function	**✓**	**✓**	**✓**	Pulmonary function
	Gastrointestinal outcomes	Digestive function	-	-	**✓**	-
	Cardiac outcomes	Cardiac function	-	**✓**	-	-
	Psychiatric outcomes	General mental health	**✓**	**✓**	**✓**	Mental health
		Anxiety/worry	**✓**	**✓**	**✓**	Psychological function
		Depression/low mood	**✓**	**✓**	-	Psychological function
**Life impact**	Global quality of life	Health-related quality of life	-	**✓**	-	Health-related quality of life
	Functioning **–** social functioning	Loneliness	**✓**	**✓**	**✓**	Social functioning
		Social connectedness	**✓**	**✓**	**✓**	Social functioning
		Peer acceptance	**✓**	**✓**	**✓**	Social functioning
		Social participation	**✓**	**✓**	**✓**	Leisure activity functioning. Participation
	Functioning – role functioning	Ability to engage in sports	**✓**	**✓**	**✓**	Leisure activity functioning
		Ability to independently participate at school	**✓**	**✓**	**✓**	Workability
		Able to maintain a safe pregnancy in the future	**✓**	-	-	-
		School/work absence	-	**✓**	**✓**	-
		Ability to engage in chosen career	-	**✓**	-	-
		Impact on educational attainment	-	-	**✓**	Workability
	Functioning – physical functioning	Activities of daily living	**✓**	**✓**	**✓**	Physical functioning
		Physical activity	-	**✓**	-	-
		Sleep	-	**✓**	-	Sleep function
	Functioning – emotional functioning/wellbeing	Self-esteem	**✓**	**✓**	**✓**	Psychological function, subdomain; self-esteem
		Body image	**✓**	**✓**	**✓**	Psychological function, subdomain; body-image
		Satisfaction with shape of back	**✓**	**✓**	-	Satisfaction, subdomain; with cosmetic result
		Satisfaction with shape of back for parent	-	-	**✓**	-
		Satisfaction with posture	**✓**	-	-	Satisfaction, subdomain; with cosmetic result
		Comfortable wearing desired clothes	**✓**	**✓**	-	-
		Ability to cope	**✓**	-	**✓**	-
		Happiness	-	**✓**	-	-
	Delivery of care	Continuity of care	**✓**	**✓**	**✓**	Satisfaction, subdomain; with treatment
		Meeting expectations of what treatment involves for patient	**✓**	**✓**	**✓**	-
		Meeting expectations of what treatment involves for parent	-	**✓**	**✓**	-
		Treatment satisfaction for patient	**✓**	**✓**	**✓**	Satisfaction, subdomain; with treatment
		Treatment satisfaction for parent	-	**✓**	**✓**	-
		Treatment satisfaction for clinical team	**✓**	**✓**	-	Surgeon’s satisfaction Environmental factors- appearance
		Satisfaction with treatment wait time for patient	**✓**	**✓**	-	Satisfaction, subdomain; with treatment
		Satisfaction with treatment wait time for parent	-	**✓**	**✓**	-
		Parent trust in clinical team	-	-	**✓**	-
		Healthcare provider communication	**✓**	**✓**	**✓**	Satisfaction, subdomain; with treatment
		Satisfaction with verbal information provision for patient	**✓**	**✓**	-	Satisfaction, subdomain; with treatment
		Satisfaction with verbal information provision for parent	-	-	**✓**	-
		Satisfaction with written information provision for patient	**✓**	**✓**	-	Satisfaction, subdomain; with treatment
		Satisfaction with written information provision for parent	**✓**	-	**✓**	-
		Satisfaction with physiotherapy support	**✓**	-	-	Satisfaction, subdomain; with treatment
		Satisfaction with psychological support	**✓**	**✓**	**✓**	Satisfaction, subdomain; with treatment
		Satisfaction with peer support	**✓**	**✓**	-	-
		Access to multi-disciplinary team	-	**✓**	**✓**	-
		Access to adolescent units	-	**✓**	-	-
		Parental anxiety/worry	**✓**	**✓**	**✓**	-
		Parental low mood/depression	-	-	**✓**	-
**Bracing only outcome domains**						
**Life impact**	Delivery of care	Ability to sleep wearing brace	**✓**	-	**✓**	N/A
		Physical restrictions when wearing brace	-	**✓**	-	N/A
		Pain or discomfort wearing brace	**✓**	**✓**	**✓**	N/A
		Pulmonary function wearing brace	-	-	**✓**	N/A
		Subtlety of brace under clothes	**✓**	**✓**	-	N/A
		Satisfaction with style of brace	-	**✓**	-	N/A
		Adherence to brace wearing	**✓**	**✓**	**✓**	N/A
		Self-efficacy to brace wearing	**✓**	**✓**	**✓**	N/A
		Clinician belief in treatment efficacy	-	-	**✓**	N/A
		Ability to engage in sports while undergoing bracing	**✓**	**✓**	**✓**	N/A
		Peer acceptance while wearing brace	**✓**	**✓**	**✓**	N/A
		Self-confidence while wearing brace	**✓**	**✓**	-	N/A
		Loneliness while brace wearing	-	**✓**	-	N/A
		Parental belief in treatment efficacy	-	**✓**	**✓**	N/A
**Resource use**	Need for further intervention	Surgery	**✓**	**✓**	**✓**	N/A
**Surgery only outcome domains**						
**Physiological/clinical**	Musculoskeletal and connective tissue outcomes	Symmetry	-	**✓**	-	-
		Spinal rotation	-	**✓**	-	Range of motion
	General disorder	Post-surgical pain	**✓**	**✓**	**✓**	Pain interference
		Post-surgical fatigue	**✓**	-	-	-
	Respiratory, thoracic and mediastinal outcomes	Post-surgical pulmonary function	**✓**	**✓**	**✓**	Pulmonary function
**Life impact**	Functioning – physical functioning	Post-surgical flexibility	**✓**	**✓**	**✓**	Range of motion
		Post-surgical ability to sleep	**✓**	-	-	Sleep function
		Post-surgical core strength	-	**✓**	-	Muscle strength
		Post-surgical recovery time	**✓**	**✓**	**✓**	-
		Post-surgical mobility	**✓**	**✓**	-	Ambulatory status
		Wound management	-	**✓**	-	-
	Functioning **–** role functioning	School/work absence	**✓**	-	-	-
		Impact on educational attainment	**✓**	-	-	-
		Engagement in contact sports	-	**✓**	-	Leisure activity functioning
	Functioning – emotional functioning/wellbeing	Self-confidence regarding scarring	**✓**	**✓**	**✓**	-
	Delivery of care	Satisfaction with post-discharge care	**✓**	-	-	Satisfaction, subdomain; with treatment
		Satisfaction with prehabilitation	-	**✓**	-	Satisfaction, subdomain; with treatment
		Satisfaction with post-surgical physiotherapy support for parent	-	-	**✓**	-
		Surgery proceeding	-	**✓**	-	-
**Adverse events**	Adverse events	Post-surgical complications	**✓**	**✓**	**✓**	Adverse events
**Resource use**	Hospital use	Number of days in hospital	**✓**	-	-	Length of hospitalization
	Need for further intervention	Repeat surgery	-	-	**✓**	Reoperations
		Readmission	**✓**	-	-	30-day readmission

* This systematic review identified randomized trials, non-randomized trials, and observational cohort studies for patients undergoing surgery only.

AIS, adolescent idiopathic scoliosis; AYAs, adolescents and young adults; COS, core outcome set; HCP, healthcare professional; N/A, not applicable.

**Fig. 1:**
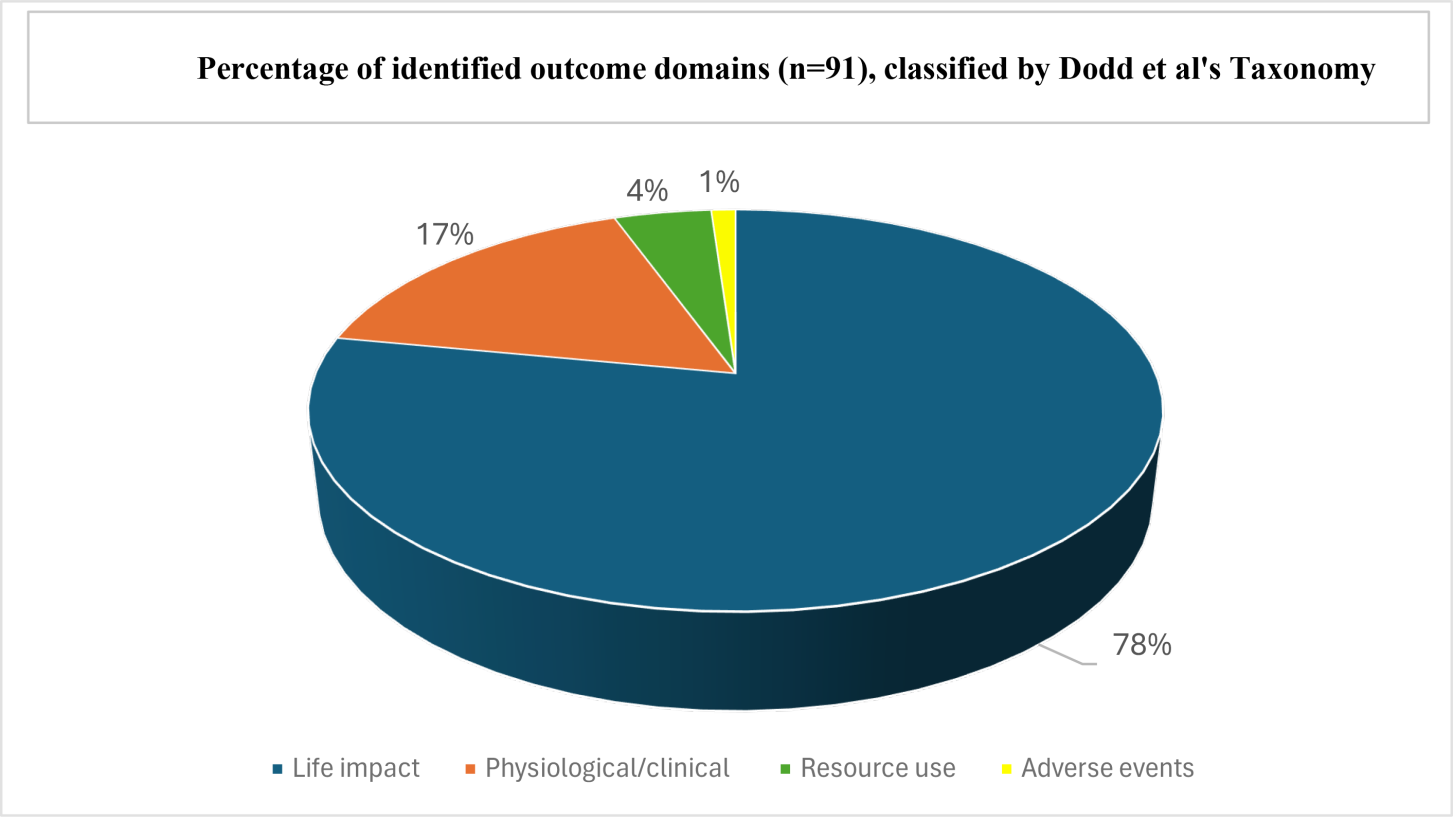
Percentage of identified outcome domains (n = 91) classified by Dodd et al’s^26^ taxonomy for classification of outcome domains in medical research.

In relation to Dodd et al’s^26^ taxonomy for classification of outcome domains, for the 53 outcome domains applying to both treatment types, the majority of identified outcomes relate to ‘life impact’ (n = 43, 81%) and a smaller proportion are classified as physiological/clinical outcomes (n = 10, 19%) (Figure 1). For the 15 additional outcome domains identified for bracing only, the majority (n = 14, 93%) relate to ‘life impact’ and one outcome relates to ‘resource use’ (surgery) Lastly, for the 23 outcome domains identified for surgery only, the majority (n = 14, 61%) relate to ‘life impact’, with a smaller number relating to physiological/clinical outcome domains (n = 5, 22%), to resource use (n = 3, 13%), and one relating to adverse events (post-surgical complications). The three participant groups identified a similar proportion of outcome domains relating to the taxonomy core areas. For example, between 75% and 78% of the outcome domains identified by each group related to ‘life impact’ and 18% to 19% of the outcome domains identified by each group are classified as physiological/clinical outcomes.

As seen in Table IV, the outcome domains identified from a systematic review (which focused on surgery only) and included in the previous COS can be mapped to some degree against 29 of 53 outcome domains (55%) applying to both treatment types, and against 14 of 23 outcome domains (61%) applying to surgery only.

In Table V, a participant quotation from the qualitative interviews is aligned to selected outcome domains to evidence the coding process.

**Table V.** Participant quotations aligned to selected outcome domains.

Dodd’s et al^26^ taxonomy core area	Example participant quotation
**Physiological/clinical**	**Curve progression:** “Well they looked at the curve first, and then they were always every appointment that always asked me if I was happy with it”. *(Participant 38, AYA)* **Pain:** “It's like, it's just really, really painful to be honest like physically, it's like you've constantly this ache. Like your back, you're never comfortable, you're always uncomfortable.” *(Participant 3, AYA)* **Fatigue:** “I think for the second year of his GCSE that’s when the effect was more for him and his tiredness and I think with work that he was submitting probably was more the issue, you know, because in the evenings, he was getting a lot of things and he was just very, very tired.” *(Participant 1, Parent)* **Pulmonary function**: “Well he plays a lot of football so he himself had noticed that he was getting very out of breath during a match, you know he hadn’t the same stamina. He would have tired after about half an hour.” *(Participant 33, Parent)* **Anxiety/worry:** “Like it was really scary. It was a really scary time for me because I was just like, wondering what was going on because I was still like finding out stuff about myself and like how I'm going to like deal with like, my scoliosis mentally first.” *(Participant 28, AYA)*
**Life impact**	**Health-related quality of life**: “So functionally, what they're able to do, and that they're able to do everything they wanted in their life, be that play sport to a high level, or just being able to go to the shops. But some people, some people are different than others, some people maybe that’s more important, but other people are really happy if they're able to do everything they need to do”. *(Participant 17, HCP)* **Peer acceptance:** “People can be very like; she has something wrong with their body. Don't want to be around someone like that. And then there's some people that are like, really welcoming.*” (Participant 28, AYA)* **Ability to engage in sports:** “Oh, I think just there's certain there are certain confidence levels, which impacts on everything, their social abilities, their work abilities, I suppose sports as well.*” (Participant 14, HCP)* **Activities of daily living**: “Just back to normality again.” *(Participant 16, HCP)* **Physical activity:** “So, getting them to start to do some sort of physical activity that they enjoy that they've got some interest in and that you think they're going to be motivated to continue.” *(Participant 19, HCP)* **Body image:** “It wasn't affecting me. But then I did as I started to grow, I started to like, get a bit insecure about my body a wee bit because I started to notice that I looked different than all these other girls in school and obviously.” *(Participant 39, AYA)* **Satisfaction with shape of back for parent:** “How she looks from the front is normal. And so actually, her back’s not awful.” (Participant 29, Parent) **Happiness:** “But you know, like, if they're not happy, then it hasn't been a success”. *(Participant 22, HCP)* **Treatment satisfaction for patient:** “I think, to me, it is just that they've had a reasonable experience in hospital for their surgery, whatever that may mean, and you know, that they're happy with the outcome and that, yes, whatever symptoms they had preoperatively are better or completely resolved, you know? so it is, it's very woolly and not very good. You know as a clinician you like you like something very defined as an endpoint, because it's, you know, it's easier to measure.” *(Participant 22, HCP)* **Satisfaction with treatment wait time for patient:** “But obviously, the most glaringly obvious is the timing and for the length of time that they have to wait to see us and the length of time, you have to wait for their surgery.” (Participant 25, HCP) **Healthcare provider communication:** “He made me feel really comfortable. He sort of came spoke to came and spoke with me after and he made it so much better. Like if I'd had if Id of had a surgeon who wasn't like as likable and charming as he was, I think I would have found it a lot more scary, whereas I felt kind of comfortable with him having done it”*. (Participant 7, AYA)* **Satisfaction with verbal information provision for patient:** “And I just haven't had the chance to be educated on that. That’s partly my own fault for not doing the research but it maybe would be nice to have someone you could talk to.” *(Participant 27, AYA)* **Satisfaction with psychological support:** “I was going to counselling with (specialist mental health support), but…I wasn't honest with them. Because I just felt like, oh, this is a new person, like I can’t trust them. And then there was that was one of the like therapists, and then I got a new one and I started to trust her, so I just said everything to her*”. (Participant 38, AYA)* **Parental anxiety/worry:** “So I suppose from a parent's point of view, that, you know, it's back to that thing of, you know, it's always with you, you're always worrying about how this is going to affect their life and the deterioration aspect of it is a very big, sort of overhanging, overarching concern. And you know, I suppose, that’s about the height of it really.” *(Participant 1, Parent)*
**Bracing only**	
**Life impact**	**Pulmonary function wearing brace:** *“She did at the start more for the breathing techniques more than anything else I didn't realize she had to learn to breathe in these things…she says I can’t breathe!” (Participant 13, Parent)* **Subtlety of brace under clothes:** “Even obviously you know, it would take money to innovate it like, but like a brace that was – it’s made out of plastic and the straps, if it was something made out of fabric that was able to blend in with clothing, I think. A lot of people would find it easier to wear it all the time so…” *(Participant 34, AYA)* **Satisfaction with style of brace:** “Yeah I think I mean basically it’s just down to comfort and maybe style of the brace, is maybe two things that we really need to be looking at. You know a lot of the patterns that would be on the brace are very outdated no teenagers these days,… and I'm looking going these kids are into Tiktok and Instagram they don't want groovy chick on them you know, so any wonder that they're not going to choose anything…they need to look at maybe updating some of the patterns and the styles on them that they want these kids to maybe embrace it a bit more”*. (Participant 21, HCP)* **Adherence to brace wearing:** “And then she’s like why do I have to wear this 23 hours a day? I just want to go and have the surgery, because she sees 6 weeks’ time she’s going to be flying and going to be fine – I don’t want to wear this brace I just want the surgery.” *(Participant 10, Parent)* **Self-confidence while wearing brace:** “Well, if braced a lot of them will find it particularly challenging to wear a brace for maybe 18–23 hours a day they also find it challenging because some of them will be self-conscious of wearing a brace under their clothes or having to take a brace off in front of their peers in school so a lot of them won’t wear a brace in their PE day because they don’t want to be seen to be taking the brace off.*” (Participant 15, HCP)*
**Resource use**	**Surgery:** “If it’s going to save from surgery…” *(Participant 28, AYA)*
**Surgery only**	
**Physiological/clinical**	**Post-surgical pain:** “And sort of getting the surgery like after, in the week that I was in hospital, it was really, really, really, really painful and very uncomfortable. And I've had a couple people like one of my friends and my cousin who are having the surgery soon, and I think my mum and dad sort of said you could speak to them about it.” *(Participant 7, AYA)*
**Life impact**	**School/work absence:** “So I just wanted it out of the way I was studying from, but the time it kind of got to the point of getting the operation, I was doing my A levels. And I was just really focused on that you know, as most students are, you know, what, I didn't want time away out of school.” *(Participant 8, AYA)* **Self-confidence regarding scarring:** “But my scars sort of faded and I know that that will continue to fade and I shouldn't be like, like ashamed of it. It's just quite like it's very big. It's very obvious. I do have to be aware of it whenever I'm wearing like, I wanted to wear it backless dress for my formal and now I'm sort of a bit like, oh, I don't know, if I want to because I don't think I want that to be like, people think when they see me do you know what I mean?” *(Participant 7, AYA)*
**Adverse events**	**Post-surgical complications:** “Yeah. So, he had the operation and they couldn't understand why, I mean, he in absolute agony. Yes, normally they're going to be in a lot of pain afterwards and that was helped with meds or whatever. But this was out of control pain. So, this went on, they were trying to encourage him to get out of bed and he just couldn't, he couldn't do what the physio wanted him to do. And then the wound actually started leaking blood itself. And then they knew that there was a blood clot had formed like a bubble on the spine inside and then I think about 5 days after the surgery, and he was nearly out of his lid with pain.*” (Participant 32, Parent)*
**Resource use**	**Readmission:** “Well, after I was actually let out of hospital quite quickly, because I was healing, but a couple of days later, I started to get like blurry vision and my blood levels were obviously quite low. So I had to go back in for them to check everything was okay. But apart from that everything went smoothly.” *(Participant 39, AYA)*

AYA, adolescent and young adult; HCP, healthcare professionial.

### Physiological/clinical outcome domains

Curve progression was identified as important by all participant groups, although it was frequently accompanied by discussion of the importance of the AYA’s satisfaction with treatment and shape of their back (reflected in the emotional functioning/wellbeing outcome domains), “Cobb angle being the big radiological measure that they'll use but aesthetically a child doesn't care what the Cobb angle is, they'll look in the mirror” (*Participant 20, HCP*). Pain and the impact of pain were discussed in depth by all participant groups, in relation to the frequency and level of pain and the restriction of activity due to pain or fear of pain, “I just wanted to be in less pain to be honest, you know, I as I said I was lying down a lot, I didn't really get to do anything that I wanted to so” (*Participant 11, AYA*). Three psychiatric outcome domains were identified, largely acknowledging the long-term challenges of adjusting to a diagnosis and treatment of scoliosis at a developmentally critical time, “You just think right, you got the operation. That’s great. Get better, and you’re straighter and all this. But it affected him really quite badly, where he took a nervous breakdown” (*Participant 32, Parent*). There were five outcome domains specifically for surgery, including symmetry and spinal rotation, which were only identified by the HCP participants.

### Life impact

There were nine important outcome domains identified relating to role functioning. The AYA’s ability to independently participate at school with AIS/through treatment (with appropriate support) was discussed, and for the HCP and parent participants, the impact of AIS/treatment on school/work absence, educational attainment and ability to engage in a chosen career, “They very openly tell you in clinics they lost friendships, not able to take part in activities, and find schoolwork quite hard. Or school time quite hard” (*Participant 25, HCP*). For AYAs, school/work absence and impact on educational attainment were only identified as important in relation to surgery itself. There were nine outcome domains identified relating to emotional functioning/wellbeing. This included the importance of self-esteem and body image (and self-confidence related to scarring for surgery specifically), and at a more granular level the AYA’s ability and confidence to wear the clothes they wish, “If people are very aware of their curve and how it looks in certain clothes or maybe in swimwear or things, that would probably be the next important thing for me” (*Participant 27, AYA*).

Significantly, there were 39 important outcome domains identified relating to delivery of care. This included outcome domains related to meeting expectations of what the treatment involves for both the patient and parent, as well as treatment satisfaction for the AYA, parent, and clinical team, “And I suppose that’s what we've been trying to do… how can we prepare kids specifically for surgery? You know… so that they know what to expect and try and do as much as we can to help anxiety and everything post operation” (*Participant 17, HCP*). There were 14 outcome domains within delivery of care specifically related to bracing. This included adherence to brace wearing as an outcome, but also a variety of outcome domains which are related to adherence, such as self-efficacy to brace wearing, peer acceptance while wearing brace, and pain or discomfort wearing brace.

### Resource use and adverse events

There were four important outcome domains identified related to resource use, with one relating to bracing only and three relating to surgery only. The need for surgery was identified for bracing as an outcome, by all participant groups, “Ideally, we don’t want the kids to have surgery I’d prefer to get them to the end and the brace has done its job that they are pain free, happy, healthy, and functioning without the need for any further treatment. That is the goal I think for everybody” (*Participant 18, HCP*). For surgery only, outcome domains related to number of days in hospital, readmission, and repeat surgery. There was also an adverse events outcome domain identified for surgery only by all participant groups; post-surgical complications, “Getting the far side of an operation with no surgical complications a lot of them would be relieved by, a lot would consider that successful” (*Participant 15, HCP*).

## Discussion

This is the first study worldwide to explore which treatment outcomes are important to adolescents with AIS, their parents, and HCPs. In doing so, it makes a novel contribution to the small body of research involving adolescents in developing COS for paediatric conditions^27^. The study highlights the ability of adolescents with AIS to identify and discuss important treatment outcomes, with a YPAG providing meaningful patient and public involvement (PPI) input into research design and conduct. This underscores the value of integrating patient perspectives in AIS research where their voice, with exception,^28^ has largely been absent.

The findings suggest that the treatment outcomes important to AYAs with AIS, their parents, and HCPs are often not prioritized for measurement in clinical trials. Most outcomes identified in this study (75% to 80%) focus on life impact, with only 18% to 19% classified as physiological or clinical. In contrast, trials evaluating AIS surgery and bracing use radiological measures, comprising 50% to 57% of outcomes measured.^9^ Additionally, 33 domains identified in this study, spanning both treatment types or surgery-specific outcomes, were absent from the systematic review of surgery research that informed the previous COS.^13^ Identifying important outcome domains is crucial for selecting measures that effectively capture treatment responsiveness. This study reinforces the need for greater use of PROMs in AIS research,^10^ especially as AYAs undergoing conservative treatment often report perceived improvements, such as enhanced self-image, even without significant change in Cobb angle.^29^

Research with UK spinal HCPs highlights limited use of outcome measures in clinical practice, with issues around perceived content and value of PROMs.^30^ Challenges with spinal registry data collection^31^ may also reflect issues with patient acceptability of current measures. On a promising note, this study shows reasonable alignment between outcomes important across stakeholder groups, and provides a list of important outcome domains with a high level of granularity. These findings align with qualitative research^32^ showing key aspects of an adolescent’s experience, such peer acceptance, are often missed by widely used measures such as the Scoliosis Research Society-22r. Findings also reflect longer-term treatment outcomes (e.g. related to future reproductive health and future career), which align to calls to consider how best to meet the needs of patients across the life course.^33^

Many outcome domains identified in this study relate to PROMs assessing treatment satisfaction and patient-reported experience measures (PREMs) measuring perceptions of care received. The focus on delivery of care was particularly pronounced for treatment outcomes identified for bracing only. This likely reflects the need for more evidence-based approaches to address patient barriers to bracing adherence,^34^ of central importance to achieving treatment outcomes for this cohort.^35^ For adolescents undergoing surgery, the emphasis on delivery of care outcome domains may support the transition towards enhanced recovery after surgery protocols.^3^

The study has several strengths, including recruitment from a regional scoliosis service, which enabled inclusion of participants from a broad geographical area encompassing both rural and urban locations and areas with differing socioeconomic profiles. The sample (n = 40), stratified into three groups, was considered sufficient based on information power,^36^ given the focused study aim, specificity of the participant group, and richness of data generated. Limitations include the use of a single-item question for sex only (we did not collect biological sex at birth; see Ashby and Haddad^37^ for discussion), the absence of participants with complications or revision surgery who may have different perspectives, and the lack of ethnic minority participants, which may limit transferability to more diverse populations. It must be considered that data were collected from one region and waiting times for treatment and service organization vary across the UK. However, this is unlikely to have influenced the range of outcomes identified, as all outcomes reported were included.

Through qualitative research, this study identifies a long list of treatment outcome domains that are meaningful to the clinical population, but many of these are not currently measured in clinical trials or integrated into spinal registries. In the shorter term this long list, alongside greater integration of patient and public involvement, will be useful to inform decision-making around choice of treatment outcomes in AIS research and service delivery. Secondly, findings indicate there is a need to more closely examine the content validity of existing outcome measures, aligned to the recent work of researchers,^32^ to ensure outcome domains are being measured in ways that are meaningful for the patient. Lastly, there is a notable lack of PREMs which have been developed or validated for use with AIS populations. Given the predominance of delivery of care outcome domains identified in the current study, this would suggest PREMs to be an important area for future AIS research.

This research study has been conducted as part of a larger initiative to develop an internationally applicable, person-centred COS for AIS treatment. The findings provide a robust foundation for an international consensus process to agree a small number of core outcomes for consistent measurement in AIS clinical trials and routine practice. Once developed, a COS for AIS treatment will focus efforts to promote the use of psychometrically robust measurement tools and encourage the development of new measures where needed, including cross-cultural validation. A well-developed COS will optimize outcome measurement, addressing challenges in AIS trials^2-6^ and embedding patient consensus in spinal registries, a hallmark of successful registries.^38^ Of importance, a COS for AIS treatment has the potential to facilitate high-quality meta-analyses of international clinical trials and the aggregation of routinely collected data. This is key to resolving existing clinical uncertainties for AIS.

**Take home message**

- When evaluating treatment (surgery or bracing) for adolescent idiopathic scoliosis, there is currently insufficient understanding of which outcomes are considered important by adolescents, their parents, and healthcare professionals.

- This study identified 91 treatment outcome domains, considered important from the perspective of young people with adolescent idiopathic scoliosis, their parents and healthcare professionals. The majority of important outcome domains related to ‘life impact’, and were identified by more than one stakeholder group suggesting a level of agreement.

- Many outcome domains considered important are not routinely being measured in clinical trials or by spinal registries. The current study findings can be used to inform the development of a person-centred core outcome set for adolescent idiopathic scoliosis treatment.

## Data Availability

The datasets generated and analyzed in the current study are not publicly available due to data protection regulations. Access to data is limited to the researchers who have obtained permission for data processing. Further inquiries can be made to the corresponding author.
